# Perceived efficacy of existing waterpipe tobacco warning labels versus novel enhanced generic and waterpipe-specific sets

**DOI:** 10.1371/journal.pone.0255244

**Published:** 2021-07-27

**Authors:** Aya Mostafa, Moustafa El Houssinie, Rasha Saad Hussein

**Affiliations:** Department of Community, Environmental, and Occupational Medicine, Faculty of Medicine, Ain Shams University, Cairo, Egypt; Manipal Academy of Higher Education, INDIA

## Abstract

**Introduction:**

Since 2008, Egypt has four existing generic bi-annually rotating warning labels (WLs) on 50% of the waterpipe tobacco packs (WTPs). The Ministry of Health Tobacco Control Unit proposed increasing WL size to 80%, removing colours and flavour imagery from WTPs, and plain packaging to help curb the rising epidemic of waterpipe tobacco smoking. Therefore, we measured the perceived efficacy of existing against novel enhanced (generic and waterpipe-specific) WTP WLs and the associated factors among Egyptian waterpipe smokers and nonsmokers.

**Methods:**

A purposive quota sample of 2014 adults was surveyed in two rounds using face interviews. At each round, participants were randomly shown one of four existing WLs, then one of four novel WLs. Participants rated the perceived efficacy of existing and novel WLs regarding the salience, depth of processing, affective reactions, credibility, relevance, perceived harm and perceived behavioural control. Data were analysed using Generalized Estimating Equations.

**Results:**

Participants rated novel WTP WLs with higher mean perceived efficacy scores than existing WLs for all measures, although both sets collectively scored modestly (59.7; 95% CI: 58.9–60.5 vs 53.0; 95% CI: 52.1–54.0, respectively; p<0.001). Relative to the existing WTP WLs, novel WLs were particularly able to induce higher salience, affective reactions, and depth of processing. Relative to the generic novel WTP WLs, waterpipe-specific WLs induced higher relevance, perceived harm, and affective reactions. Nonsmokers scored higher than waterpipe tobacco smokers, specifically for perceived behavioral control (65.0±32.5 vs 43.6±19.8, respectively; p<0.001). WTP WLs featuring proximal risks, such as dental effects (β = 9.70; 95% CI: 7.00–12.40), fetal harm (β = 9.42; 95% CI: 6.75–12.10), or toxic contents (β = 9.14; 95% CI: 6.58–11.70) were strongly associated with participants’ perceived efficacy scores. Among other independent factors, rural residence (β = 24.09; 95% CI: 22.21–25.97), being a nonsmoker (β = 10.51; 95% CI: 8.92–12.10), survey round 2 (β = 6.96, 95% CI: 5.73–8.19), the novel WTP WL set (β = 6.68; 95% CI: 6.19–7.17), and having higher education (β = 6.31; 95% CI: 4.34–8.27) were highly associated with participants’ perceived efficacy scores.

**Conclusions:**

Waterpipe-specific WLs on plain WTPs that feature proximal risks and address different population subgroups need to be developed in conjunction with awareness raising campaigns on WTS harms to reinforce the credibility of WTP WLs. Our findings suggest the proposed WTP WL enhancements by the Tobacco Control Unit may support a more effective WTP labelling policy within a comprehensive waterpipe-specific tobacco control framework.

## Introduction

Waterpipe tobacco smoking (WTS) is associated with periodontal, pulmonary, and cardiovascular diseases; cancers of the mouth and lung; low birth weight; various toxic content exposures; and addiction [1–3]. Despite the growing evidence of its adverse health effects, WTS has gained social acceptance because of misconceptions regarding its harms [4]. Tobacco control programmes should upscale public education efforts and communicate these WTS health effects more effectively, particularly in countries with high WTS prevalence rates [2, 5].

Past 30-day WTS rates in youth reached 11.4%, 22.7% and 37.2% in some reports from the United States, Latvia, and Lebanon, respectively [6]. In Egypt, one of the largest waterpipe tobacco markets in the world [7], up to 25.4% of private university students reported WTS [8]. National estimates of current WTS in Egyptian males and females aged 15–69 years old were 8.7% and 0.1%, respectively [9].

Applying textual and pictorial warning labels (WLs) on tobacco products is a cost-effective method of increasing public awareness to health-related smoking dangers, increasing the likelihood of quitting among smokers, and deterring smoking initiation among nonsmokers [10, 11]. Viewing WLs mediate these outcomes through policy-specific measures (such as salience and depth of processing) and general measures (such as perceived risk, affective reactions, credibility, relevance, self-efficacy) that result in quit intentions or avoidance, which in turn affect the smoking behavior [12]. Based on behavioural theories, these measures have been organized within conceptual frameworks of WL impact [11–14].

In line with this evidence, Article 11 of the World Health Organization Framework Convention on Tobacco Control (WHO FCTC) guidelines recommend large on-pack pictorial WLs and plain packaging [15, 16]. These WL enhancements could more effectively reduce smoking through rendering tobacco packaging and smoking less tempting, maximizing warning salience, and reducing misperceptions about tobacco use harm [17–20], especially in nonsmokers and non-established smokers [21]. This evidence is principally from cigarette studies; little is known about such effects on non-cigarette tobacco use, particularly WTS [22, 23].

Studies that quantitively examined the impact of enhanced WTP WLs are scarce. Three surveys in Canada, the United States, and Jordan examined hypothetical warnings shown virtually on computer screens (text-only versus pictorial WLs) [24–26]. Pictorial WLs in these studies had modest effects on established waterpipe smokers. However, the impact of such WLs on nonsmokers was not assessed.

Egypt implemented textual WLs on tobacco products in 1981 and introduced pictorial WLs in 2008 after ratifying the WHO FCTC in 2005 [27]. A set of four generic pictorial WLs and accompanying text, besides an additional standard textual warning: “smoking damages health and causes death”, is applied to both cigarette and waterpipe tobacco packs (WTPs) and is rotated every 2 years. These WLs cover 50% of the front and back surfaces of WTPs and carry the quitline number [27]. However, existing tobacco packs depict colourful fruits and flavours in brand imagery [27]. In response to the rising WTS rates in Egypt, the Ministry of Health Tobacco Control Unit proposed in 2015 amending the WL regulations by introducing three changes: increasing the size of WLs to 80% of the pack surface, removing colours and flavour imagery, and applying plain packaging.

**Fig 1:**
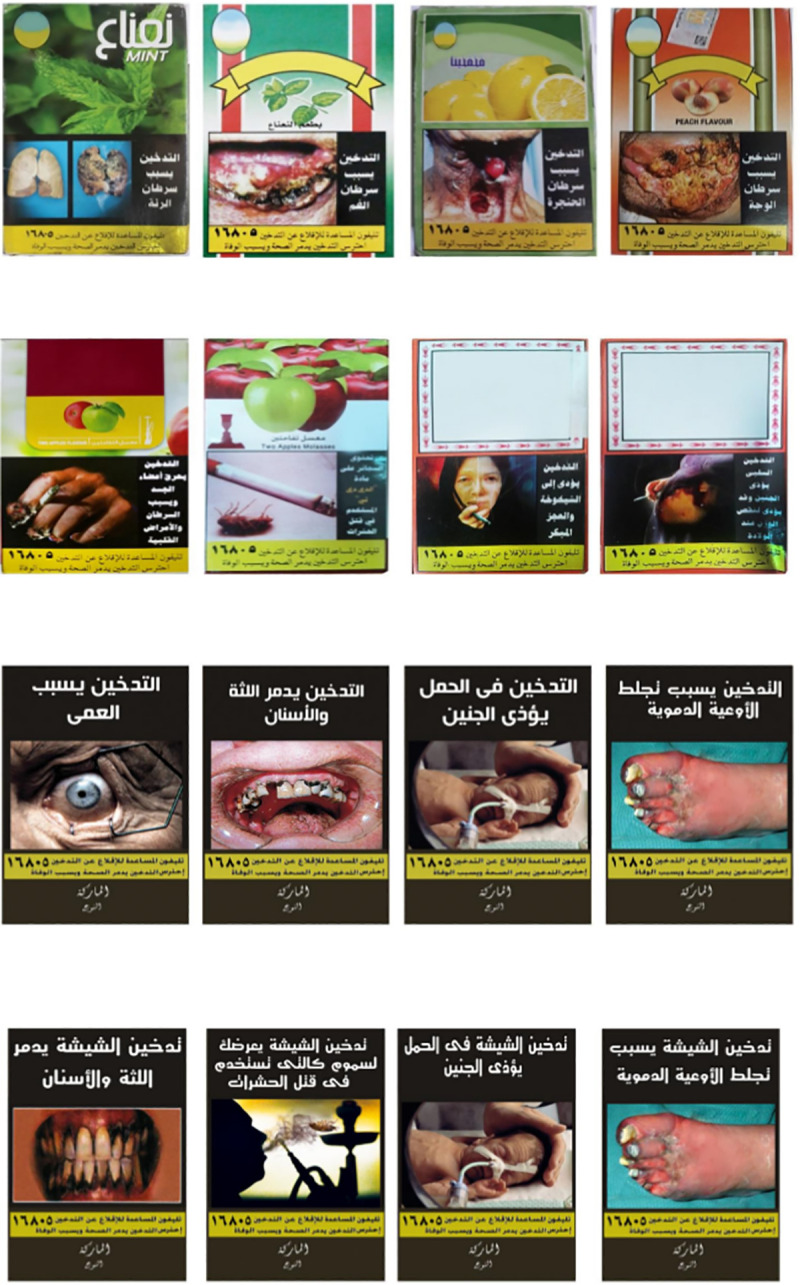
Existing and novel WTP WLs in survey rounds 1 and 2. 1.1 Existing WTP WLs in the Egyptian market during survey round 1. Textual warnings read: Smoking causes. (A) Lung cancer (B) Mouth cancer (C) Throat cancer (D) Face cancer. 1.2 Existing WTP WLs in the Egyptian market during survey round 2. Textual warnings read: (A) Smoking burns body organs and causes cancers and cardiac diseases (B) Cigarettes contain “DDT” that is used in killing insects (C) Smoking leads to early aging and disability (D) Passive smoking harms the fetus and may lead to reduced weight at birth. 1.3 Novel WTP WLs with generic text used during survey round 1. Textual warnings read: Smoking. (A) causes blindness (B) destroys teeth and gums (C) in pregnancy harms the fetus (D) causes blood vessel clotting. 1.4 Novel WTP WLs with waterpipe-specific text used during survey round 2. Textual warnings read: (A) Waterpipe smoking destroys teeth and gums (B) Waterpipe smoking exposes you to toxins like that used to kill insects (C) Waterpipe smoking during pregnancy harms the fetus (D) Waterpipe smoking causes blood vessel clotting. Reprinted under a CC BY license, with permission from the WHO, original copyright [2014–2016].

To provide preliminary insights into the potential effects of this policy approach, the authors collaborated with the Tobacco Control Unit and developed novel enhanced WLs (generic and waterpipe-specific) that address the three changes proposed in the WL regulation amendments. Each novel set comprised four WLs applied to actual WTPs. Novel WTP WLs carried different textual and pictorial content than the existing sets (**Fig 1**). The generic novel WL set was examined in a qualitative study [28, 29]; the results of which were used to design the waterpipe-specific novel WL set. Details of development of the novel WLs were described elsewhere [28, 29].

To complement the authors’ qualitative findings in informing WTP labelling policy, we quantitatively examined waterpipe smokers’ and nonsmokers’ perceived efficacy of both existing and novel enhanced generic and waterpipe-specific sets in two survey rounds—based on the theory of planned behaviour [30]. In this article, we present how participants rated the perceived efficacy and its subscale measures after viewing one of the four existing and one of the four novel WTP WLs at each survey round. The perceived efficacy subscale measures included salience, credibility, relevance, perceived harm, affective reactions, depth of processing, and perceived behavioural control. We also examined the background characteristics associated with participants’ perceived efficacy of existing and novel WTP WL sets.

## Subjects and methods

### Ethics statement

This study was approved by the Ethical Review Committee of Faculty of Medicine, Ain Shams University (FMASU R 10/2015 and 10a/2016). Participation was voluntary. All participants provided verbal and suitably informed consent. Data were collected anonymously, and confidentiality was ensured. More details about the consent process have been described previously [23].

### Study design and procedures

The current study was part of a larger study examining the impact of WTP WLs on Egyptian waterpipe smokers and nonsmokers. Details of the larger study design, sampling method, sample size, and survey administration have been detailed previously [3, 23, 31]. The larger study consisted of two rounds of identical cross-sectional surveys conducted during 2015–2017. The target sample size was 1025 participants at each survey round (932 plus an additional 10% to account for missing observations). We recruited 2014 (1015 in survey round 1 and 999 in survey round 2) waterpipe smokers and nonsmokers residing in rural and urban regions of Egypt via purposive quota sampling. Males and females ≥18 years were invited to complete a face-to-face interview questionnaire after obtaining informed consent. In this article, we report details of the current study design and tools.

The conceptual framework for the current study was based on the theory of planned behaviour [12, 30]. Within the larger cross-sectional comparative study, we explored participants’ perceived efficacy of WTP WLs—with respect to salience, credibility, relevance, perceived harm, affective reactions, depth of processing, and perceived behavioural control *—*after viewing one of the four existing and one of the four novel WTP WLs at each survey round.

The existing WTP WLs in survey round 1, which occurred from July through November 2015, were different from those in survey round 2, which occurred from September 2016 through January 2017. The study comprised two survey rounds, so we could assess two different sets of existing versus two different sets of novel WTP WLs; each set comprised four WTP WLs.

The same procedures were applied in both survey rounds, where the study tools consisted of a face-to-face interview questionnaire plus existing and novel WTP WLs. Participants in survey round 1 were different from participants in survey round 2. At each survey round, the interviewers held one survey questionnaire and two opaque bags during each interview, a bag for the four existing and another for the four novel WTP WLs. Both existing and novel WTP WLs were shown to participants on actual WTPs to provide a more realistic experience. After completing data on the participants’ background characteristics and history of WTS, the interviewer randomly took out one existing WTP WL from among the four available for that set. All participants were asked to closely examine the presented WTP WL for a minute and rate its perceived efficacy. Then, the interviewer repeated the same procedure at random with one of the four novel WTP WLs. This design was observational; it was not intended as an experimental study (see limitations). Each of the four existing or four novel WTP WLs for that survey round were used at an approximately equal rate. In both survey rounds, all participants rated the perceived efficacy of an existing WL first before exposure to a novel WL (see limitations).

### Study tools

#### Existing and novel WTP WLs

The two existing and two novel WTP WL sets used in the current study (four WLs in each set, i.e. 16 WLs in total) are displayed and described in **Fig 1**. Details of the existing WTP WLs are provided in the introduction section and have been previously described [23]. Pictures and text displayed in the four existing WTP WLs during survey round 2 depicted cigarettes, although they were employed on WTPs. Details of the process of why and how novel WTP WLs have been developed and selected were reported previously [28, 29]. We designed two sets of novel WTP WLs with enhanced size, text, imagery, and plain packaging based on the proposal of the Egyptian Ministry of Health Tobacco Control Unit, the available evidence on WTS health outcomes [32], the WHO FCTC recommendations for plain packaging [16], and our qualitative study that was conducted in parallel to survey round 1 [28]. Thus, each novel set comprised four WTP WLs that were applied to actual WTPs, including a picture, text and the quitline number, where we increased the size of novel WLs to cover 80% of the upper front and back WTP display areas against a dark plain background and did not list flavours or include figurative signs. The novel WTP WLs used in survey round 1 carried generic text, while those used in survey round 2 carried waterpipe-specific text **Fig 1**.

#### Survey questionnaire measures

Details of the survey questionnaire development, pilot testing and administration were reported previously [23]. The survey questionnaire included several sections; we focus in this article on presenting measures related to perceived efficacy of WTP WLs.

Data for the current study included:

a) background characteristics: age, gender, urban/rural residence, education, occupation, marital status, and exposure to secondhand smoke at the household.

b) history of tobacco use: for the current study, we considered WTS and cigarette smoking. Participants were defined as current “waterpipe smokers” if they reported WTS at least once in the past 30-days; otherwise, they were defined as “nonsmokers”. Here forth, “nonsmokers” refer to non-waterpipe smokers. If participants currently (i.e. in the past 30-days) smoked cigarettes ‘sometimes’ or ‘daily’ they were considered current “cigarette smokers”.

c) perceived efficacy of WTP WLs [12, 24]: was assessed via 14 individual measures for current waterpipe smokers and 12 individual measures for nonsmokers. The individual measures represent seven subscales:

- *salience* (one measure: WL grabs the participant’s attention)
- *credibility* (one measure: WL was believable)
- *relevance* (one measure: WL was relevant to the participant)
- *perceived harm* (one measure: made the participant concerned about WTS health hazards)
- *affective reactions* (four measures: WL made the participant feel surprised, frightened, unpleasant, or avoided looking at it)
- *depth of processing* (three measures: WL invites closer scrutiny, is understandable, and accurately depicts WTS health hazards)
- *perceived behavioural control* (three measures for current waterpipe smokers or one measure for nonsmokers: how likely viewing the WL would affect participants’ perceived motivation to quit WTS, perceived reduction of the number of hagars smoked, perceived forgoing a smoke, if they were waterpipe smokers, and perceived deterring from initiating WTS, if they were nonsmokers).

Internal reliability of the seven subscales composing the perceived efficacy measures was examined using Cronbach’s alpha: α = 0.924; composite measures were affective reactions (α = 0.923), depth of processing (α = 0.819), and perceived behavioural control (α = 0.911).

Participants rated the perceived efficacy of WTP WLs by answering questions on the individual perceived efficacy measures, which were scored on a Likert scale from 1 to 10, with 1 representing not at all and 10 representing very likely (**S1 Appendix**).

### Statistical analyses

Data were analysed using the Statistical Package for Social Sciences (SPSS version 25). Descriptive analyses were performed to obtain the means, standard errors or deviations, and frequencies.

The main outcome in the current study is participants’ perceived efficacy of existing and novel WTP WLs. Its total score was created as a sum of the individual measures scores with answer options ranging from 1–10, i.e. sum of 14 individual measures for current waterpipe smokers (range: 14–140) and 12 individual measures for nonsmokers (range 12–120). Participants’ total perceived efficacy percentage scores and 95% confidence intervals (CI) were calculated by dividing participants’ actual total perceived efficacy scores by the total possible score for current waterpipe smokers (140) and nonsmokers (120). The same method was applied to each of the seven subscale scores. This standardization of the perceived efficacy scores between waterpipe smokers and nonsmokers made a direct comparison of the relative importance of different subscales possible. Then, the percentage scores were used in further analyses.

Bivariate analyses were performed using independent samples t-test for continuous variables and chi-squared test for categorical variables. Paired samples t-test was conducted to compare within-subject differences in participants’ total perceived efficacy scores and subscale scores regarding the existing versus novel WTP WL sets.

Different multivariable analyses were conducted:

Generalized estimating equations (GEE) analysis was performed to account for within subject correlation and repeated measures data and to examine factors associated with participants’ total perceived efficacy scores (entered as a continuous dependent variable) of existing and novel WTP WL sets. The independent variables introduced into the GEE model included: age (continuous); gender (male, female); residence (urban, rural); education (university, lower); occupation (skilled, unskilled); marital status (married, unmarried); exposure to secondhand smoke at the household (yes, no); WTS status (current waterpipe smoker, nonsmoker); current cigarette smoking (yes, no); survey round (round 1, round 2); and type of WTP WL set (existing, novel). Linear main effects model with the normal distribution and identity link were used for this continuous outcome variable. The unstructured working correlation matrix was applied.

One-way Analysis of Variance (ANOVA) followed with post-hoc pairwise comparisons based on Bonferroni’s correction was conducted to identify significant associations among the eight WTP WLs within each of the existing and novel sets.

Two generalized linear models (GLM) were performed separately for the existing and the novel WTP WL sets (each entered as a continuous dependent variable) to identify which WTP WLs within each set as well as other factors were independently associated with participants’ total perceived efficacy scores. The same independent variables mentioned above were introduced into the GLM plus the eight WTP WLs of the corresponding set (entered as dummy variables, excluding the WTP WL that showed the least total perceived efficacy score: lung cancer from the existing WTP WL set and blindness from the novel WTP WL set).

Separate linear regression models were done for each of the following participants’ perceived efficacy subscales (each entered as a continuous dependent variable): salience, credibility, relevance, perceived harm, affective reactions, depth of processing, and perceived behavioural control. The independent variables mentioned above were entered into each model to explore the different associations with each of the perceived efficacy subscale measures of existing and novel WTP WLs.

For all analyses, p-values ≤ 0.05 were considered statistically significant.

## Results

### Sample characteristics

A total of 2014 individuals (1490 waterpipe smokers and 524 nonsmokers) participated in the study. Participants’ background characteristics are presented in **Table 1.**

**Table 1:** Background characteristics of participants by survey round, Egypt, 2015–2017 (N = 2014).

	Total	Round 1	Round 2	χ2	p-value^a^
	N = 2014	N = 1015	N = 999		
	n (%)	n (%)	n (%)		
**Age group**					
18–24	743 (36.9)	388 (38.2)	355 (35.5)	1.566	0.211
≥25	1271 (63.1)	627 (61.8)	644 (64.5)		
**Gender**					
Female	194 (9.6)	108 (10.6)	86 (8.6)	2.388	0.122
Male	1820 (90.4)	907 (89.4)	913 (91.4)		
**Residence**					
Urban	820 (40.7)	421 (41.5)	399 (39.9)	0.493	0.482
Rural	1194 (59.3)	594 (58.5)	600 (60.1)		
**Education**					
University/vocational	943 (46.8)	453 (44.6)	490 (49.0)	3.948	0.047
Less than university/vocational	1071 (53.2)	562 (55.4)	509 (51.0)		
**Occupation**					
Unskilled	987 (49.0)	559 (55.1)	428 (42.8)	30.139	<0.001
Skilled	1027 (51.0)	456 (44.9)	571 (57.2)		
**Marital Status**					
Married	1271 (63.1)	637 (62.8)	634 (63.5)	0.107	0.743
Unmarried	743 (36.9)	378 (37.2)	365 (36.5)		
**Exposure to secondhand smoke**					
No	742 (36.8)	354 (34.9)	388 (38.8)	3.396	0.065
Yes	1272 (63.2)	661 (65.1)	611 (61.2)		
**WTS status**					
Current waterpipe smoker	1490 (74.0)	706 (69.6)	784 (78.5)	20.82	<0.001
Nonsmoker	524 (26.0)	309 (30.4)	215 (21.5)		
**Cigarette smoker**					
No	1257 (62.4)	632 (62.3)	624 (62.6)	0.019	0.891
Yes	757 (37.6)	383 (37.7)	374 (37.4)		

^**a**^ Chi-squared test

### Perceived efficacy of existing and novel WTP WLs

In general, participants rated the novel WTP WLs with significantly higher overall and subscale perceived efficacy scores than the existing WTP WLs, although both sets scored modestly and the differences were not large (mean total percentage scores (95% CI) were 59.7 (58.9, 60.5) versus 53.0 (52.1, 54.0), respectively) **Table 2**. The highest perceived efficacy subscale scores were observed for the ability of both existing and novel sets to induce salience, affective reactions, and perceived harm **Table 2**.

**Table 2:** Total perceived efficacy of existing WTP WLs and subscale scores among current waterpipe smokers and nonsmokers in both survey rounds, Egypt, 2015–2017 (n = 2014).

			Existing WTP WLs		Current waterpipe smokers^b^		Novel WTP WLs		Current waterpipe smokers^b^	
					No	Yes			No	Yes
					(n = 524)	(n = 1490)			(n = 524)	(n = 1490)
Perceived efficacy	Number of items	Total possible score	Mean score (95% CI)	Percentage score (95%CI)	Mean percentage score ± SD	Mean percentage score ± SD	Mean score (95% CI)	Percentage score (95%CI)	Mean percentage score ± SD	Mean percentage score ± SD
**Total score**	14	140	71.1 (69.8, 72.4)	53.0 (52.1, 54.0)	60.6 ± 20.3	50.4 ± 22.1	80.2 (79.1, 81.2)	59.7 (58.9, 60.5)	66.1 ± 16.3	57.5 ± 18.0
	(or 12)^**a**^	(or 120) ^**a**^								
**Salience**	1	10	6.6 (6.4, 6.7)	65.6 (64.2, 67.1)	69.8 ± 32.2^**c**^	64.2 ± 34.1^**c**^	7.5 (7.3, 7.6)	74.6 (73.4, 75.9)	76.9 ± 27.6^**d**^	73.8 ± 28.4^**d**^
**Credibility**	1	10	4.2 (4.1, 4.3)	41.7 (40.9, 42.5)	47.8 ± 19.1	39.6 ± 17.6	4.7 (4.7, 4.8)	47.3 (46.5, 48.2)	52.0 ± 19.4	45.7 ± 18.7
**Relevance**	1	10	4.5 (4.4, 4.6)	45.4 (44.4, 46.4)	42.4 ± 22.2	46.5 ± 23.4	5.1 (5.0, 5.2)	51.4 (50.5, 52.4)	47.2 ± 24.2	52.9 ± 21.6
**Perceived harm**	1	10	5.1 (4.9, 5.2)	50.6 (49.5, 51.7)	60.8 ± 26.5	47.0 ± 23.8	5.7 (5.6, 5.8)	56.8 (55.8, 57.9)	66.5 ± 24.1	53.4 ± 22.0
**Affective reactions**	4	40	26.4 (25.9, 27.0)	66.0 (64.7, 67.4)	71.4 ± 26.1	64.1 ± 32.3	29.3 (28.8, 29.7)	73.2 (72.1, 74.3)	77.3 ± 21.3	71.8 ± 26.9
**Depth of processing**	3	30	14.6 (14.3, 14.8)	48.6 (47.8, 49.5)	53.8 ± 17.6	46.8 ± 19.4	16.5 (16.3, 16.7)	55.0 (54.3, 55.7)	58.9 ± 15.5	53.7 ± 16.3
**Perceived behavioural control**	3	30	9.8 (9.5, 10.0)	42.9 (41.8, 44.1)	59.8 ± 33.8	37.0 ± 20.0	11.4 (11.1, 11.6)	49.1 (48.0, 50.3)	65.0 ± 32.5	43.6 ± 19.8
	(or 1) ^**a**^	(or 10)^**a**^								

^**a**^ numbers between brackets are in the case of nonsmokers

^**b**^ Independent samples t-test. All p-values are <0.001

^**c**^ p-value = 0.001

^**d**^ p-value = 0.034

Nonsmokers reported significantly higher perceived efficacy scores for both existing and novel WTP WL sets than the WTS group regarding all subscales except for relevance. Both nonsmokers and waterpipe tobacco smokers scored similarly low for credibility and perceived behavioral control. However, the highest differences between nonsmokers and waterpipe tobacco smokers regarding the subscale scores were observed for perceived behavioral control (mean percentage scores ± SD were 59.8 ± 33.8 and 37.0 ± 20.0 for the existing WTP WLs, and 65.0 ± 32.5 and 43.6 ± 19.8 for the novel WTP WLs, respectively) **Table 2**.

Overall, the highest paired mean score differences between novel and existing WTP WL sets were observed for salience, affective reactions, and depth of processing **Table 3**. The paired mean score differences between novel and existing WTP WLs that were shown in survey round 2 were higher than those shown in survey round 1 regarding all perceived efficacy subscales except salience. Higher paired mean score differences were particularly observed for the ability of novel WTP WLs in survey round 2 to induce perceived harm, relevance, and affective reactions (paired mean differences between novel and existing WTP WLs in survey round 2 versus survey round 1 were: 7.2 vs 5.3, 7.0 vs 5.1, and 8.0 vs 6.4, respectively **Table 3**.

**Table 3:** Mean difference in participants’ perceived efficacy percentage scores of novel and existing WTP WLs by survey round, Egypt, 2015–2017 (n = 2014).

Perceived efficacy	Paired mean difference^a^	SD	SE	95% CI of the difference		t
	(Novel-Existing)			Lower	Upper	
**Total (n = 2014)**						
**Total score**	6.68	11.20	0.25	7.17	6.19	26.77
**Salience**	8.98	16.06	0.36	9.68	8.28	25.08
**Credibility**	5.65	18.87	0.42	6.47	4.82	13.43
**Relevance**	6.02	16.25	0.36	6.73	5.31	16.61
**Perceived harm**	6.26	14.86	0.33	6.91	5.61	18.89
**Affective reactions**	7.19	13.70	0.31	7.79	6.59	23.55
**Depth of processing**	6.40	13.46	0.30	6.98	5.81	21.32
**Perceived behavioural control**	6.19	12.55	0.28	6.74	5.65	22.16
**Survey round 1 (n = 1015)**						
**Total score**	6.16	10.87	0.34	5.49	6.83	18.04
**Salience**	9.81	17.53	0.55	8.73	10.89	17.84
**Credibility**	5.56	20.03	0.63	4.32	6.79	8.84
**Relevance**	5.08	18.24	0.57	3.96	6.21	8.88
**Perceived harm**	5.30	15.66	0.49	4.34	6.27	10.78
**Affective reactions**	6.40	13.84	0.43	5.55	7.25	14.74
**Depth of processing**	5.89	13.44	0.42	5.06	6.72	13.96
**Perceived behavioural control**	5.59	13.15	0.41	4.78	6.40	13.53
**Survey round 2 (n = 999)**						
**Total score**	7.22	11.51	0.36	6.50	7.93	19.82
**Salience**	8.13	14.39	0.46	7.23	9.02	17.86
**Credibility**	5.74	17.63	0.56	4.64	6.83	10.29
**Relevance**	6.97	13.90	0.44	6.10	7.83	15.85
**Perceived harm**	7.23	13.95	0.44	6.36	8.09	16.37
**Affective reactions**	7.99	13.51	0.43	7.15	8.82	18.68
**Depth of processing**	6.91	13.47	0.43	6.07	7.74	16.21
**Perceived behavioural control**	6.81	11.87	0.38	6.08	7.55	18.14

^**a**^ Paired samples t-test. All p-values are <0.001

### Comparison between different WTP WLs

**Fig 2:**
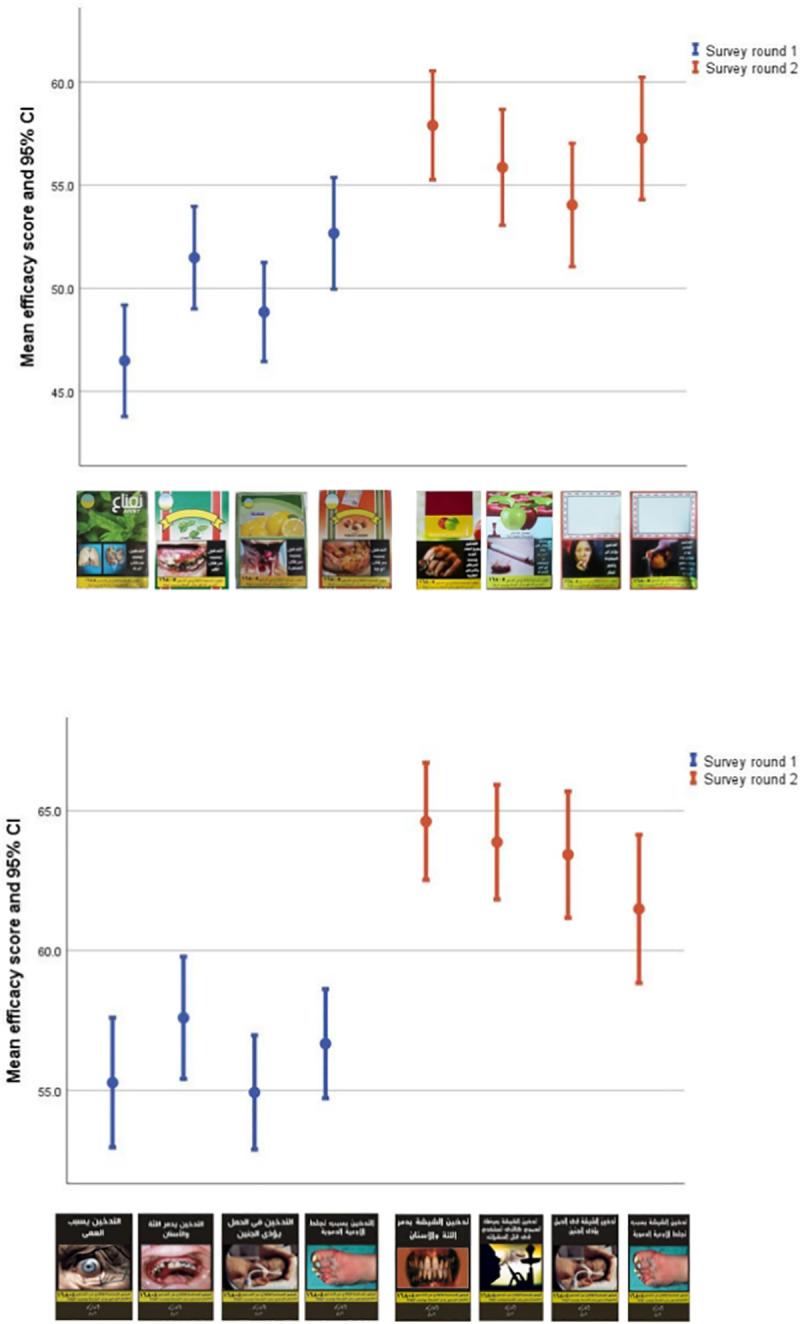
Mean efficacy scores and 95% confidence intervals of existing and novel WTP WLs in surveys round 1 (in blue) and 2 (in red). 2.1 Mean efficacy scores and 95% confidence intervals (CI) of existing WTP WLs in survey rounds 1 (in blue) and 2 (in red). 2.2 Mean efficacy scores and 95% confidence intervals (CI) of novel WTP WLs in survey rounds 1 (in blue) and 2 (in red). WTP WLs in this figure are reprinted under a CC BY license, with permission from the WHO, original copyright [2014–2016].

Usage rates of different WTP WLs in survey rounds 1 and 2 are described in **S1 Table**. Participants rated WTP WLs that were shown in survey round 2, either the existing or novel sets, with significantly higher overall perceived efficacy scores than those shown in survey round 1 **Fig 2**. Regarding the existing WTP WLs, participants reported the lowest mean perceived efficacy percentage scores for these WLs in survey round 1: “Smoking causes lung cancer” (46.5 ± 20.5) and “Smoking causes throat cancer” (48.5 ± 20.9), whereas the highest ratings were for these WLs in survey round 2: “Smoking burns body organs and causes cancers and cardiac diseases” (57.9 ± 21.1) and “Passive smoking harms the fetus and may lead to reduced weight at birth” (57.3 ± 23.2) **Fig 2.1**. For the novel WTP WLs, participants reported the lowest mean perceived efficacy percentage scores for these WLs in survey round 1: “Smoking in pregnancy harms the fetus” (54.9 ± 18.2) and “Smoking causes blindness” (55.3 ± 17.2), whereas the highest ratings were for these WLs in survey round 2:”Waterpipe smoking destroys teeth and gums and gum decay” (64.61± 16.5) and “Waterpipe smoking exposes you to toxins like that used to kill insects” (63.9 ± 18.4) **Fig 2.2**.

The individual impact of different WTP WLs on participants’ perceived efficacy scores are presented in **Table 4**. Results from the GLM confirmed that novel WTP WLs generally received significantly higher perceived efficacy scores than the existing WTP WLs, specifically those shown in survey round 2 (p<0.001). Among the existing WTP WL set, the WLs “Cigarettes contain ‘DDT’ that is used in killing insects”, “Passive smoking harms the fetus and may lead to reduced weight at birth”, and “Smoking leads to early aging and disability” had the highest significant associations with participants’ perceived efficacy scores [β = 9.25 (95% CI: 6.61, 11.89); β = 9.02 (95% CI: 6.32, 11.72); and β = 8.69 (95% CI: 5.99, 11.39), respectively]. Among the novel WTP WL set, the WLs “Waterpipe smoking destroys teeth and gums”, “Waterpipe smoking during pregnancy harms the fetus”, and “Waterpipe smoking exposes you to toxins like that used to kill insects” had the highest significant associations with participants’ perceived efficacy scores [β = 9.70 (95% CI: 7.00, 12.40); β = 9.42 (95% CI: 6.75, 12.10); and β = 9.14, (95% CI: 6.58, 11.70), respectively] **Table 4**.

**Table 4:** Generalized linear models for factors associated with perceived efficacy scores of existing and novel WTP WLs.

	Perceived efficacy of existing WTP WLs					Perceived efficacy of novel WTP WLs				
	β	95% CI		Wald Chi-Square	p-value	β	95% CI		Wald Chi-Square	p-value
**Gender** (male)	-1.67	-4.09	0.75	1.84	0.175	1.08	-1.32	3.47	0.78	0.378
**Age** (≥25)	2.63	0.64	4.63	6.71	0.010	1.52	-0.46	3.50	2.26	0.132
**Residence** (rural)	30.73	29.00	32.45	1220.47	<0.001	17.60	15.89	19.30	409.83	<0.001
**Education** (university/vocational)	5.61	3.69	7.54	32.55	<0.001	6.90	4.99	8.81	50.10	<0.001
**Occupation** (skilled)	3.17	1.36	4.99	11.77	0.001	2.92	1.12	4.71	10.12	0.001
**Marital Status** (unmarried)	4.90	2.87	6.93	22.36	<0.001	4.17	2.15	6.19	16.41	<0.001
**Exposure to secondhand smoke** (yes)	5.03	3.45	6.61	38.85	<0.001	3.39	1.82	4.96	17.95	<0.001
**Cigarette smoker** (yes)	2.44	1.05	3.82	11.84	0.001	1.20	-0.18	2.58	2.92	0.088
**WTS status** (nonsmoker)	11.63	10.04	13.22	205.39	<0.001	9.30	7.73	10.88	133.99	<0.001
WTP WLs*										
**1**	2.09	-0.51	4.70	2.49	0.115	3.30	0.55	6.04	5.54	0.019
**2**	2.21	-0.36	4.78	2.84	0.092	1.58	-0.96	4.13	1.48	0.223
**3**	2.73	0.01	5.46	3.86	0.049	2.45	-0.16	5.05	3.39	0.066
**4**	6.21	3.52	8.90	20.47	<0.001	9.70	7.00	12.40	49.64	<0.001
**5**	9.25	6.61	11.89	47.15	<0.001	9.14	6.58	11.70	49.04	<0.001
**6**	8.69	5.99	11.39	39.88	<0.001	9.42	6.75	12.10	47.78	<0.001
**7**	9.02	6.32	11.72	42.76	<0.001	8.77	5.94	11.61	36.81	<0.001

* WTP WLs

**- Existing warning labels** (1–7)

Reference (WTP WL that showed the least total perceived efficacy score): Lung cancer

(1) Smoking causes mouth cancer

(2) Smoking causes throat cancer

(3) Smoking causes face cancer

(4) Smoking burns body organs and causes cancers and cardiac diseases

(5) Cigarettes contain ‘DDT’ that is used in killing insects

(6) Smoking leads to early aging and disability

(7) Passive smoking harms the fetus and may lead to reduced weight at birth

**- Novel warning labels** (1–7)

Reference (WTP WL that showed the least total perceived efficacy score): Blindness

(1) Smoking destroys teeth and gums

(2) Smoking in pregnancy harms the fetus

(3) Smoking causes blood vessel clotting

(4) Waterpipe smoking destroys teeth and gums

(5) Waterpipe smoking exposes you to toxins like that used to kill insects

(6) Waterpipe smoking during pregnancy harms the fetus

(7) Waterpipe smoking causes blood vessel clotting

### Factors associated with perceived efficacy of WTP WLs

In the bivariable analysis, participants who were: males, younger adults (18–24 years old), rural residents, unmarried, had lower education, skilled workers, exposed to household secondhand smoke, nonsmokers, and cigarette smokers reported a significantly higher perceived efficacy score than their counterparts for both the exiting and novel WTP WLs **S2 Table**.

In the multivariable analyses, the GLM revealed statistically significant associations between participants’ perceived efficacy and all participants’ background characteristics except for gender in the model for existing WTP WLs, and except for gender, age, and cigarette smoking in the model for novel WTP WLs **Table 4**.

In the GEE model, all background characteristics were independently associated with participants’ overall perceived efficacy scores of WTP WLs **Table 5**. Adjusted for other variables in the GEE model, the following variables showed the strongest significant associations with participants’ overall perceived efficacy scores: rural residence (β = 24.09, 95% CI: 22.21, 25.97), being a nonsmoker (β = 10.51, 95% CI: 8.92, 12.10), survey round 2 (β = 6.96, 95% CI: 5.73, 8.19), the novel WTP WL set (β = 6.68,95% CI: 6.19, 7.17), and having higher education (β = 6.31, 95% CI: 4.34, 8.27) **Table 5**.

**Table 5:** Generalized estimation equation model for factors associated with perceived efficacy of WTP WLs.

	β	95% Wald CI		Wald Chi-Square	p-value
		Lower	Upper		
**Gender** (male)	-0.33	-3.52	2.86	0.040	0.841
**Age** (≥25)	2.03	-0.17	4.24	3.278	0.070
**Residence** (rural)	24.09	22.21	25.97	632.704	<0.001
**Education** (university/vocational)	6.31	4.34	8.27	39.631	<0.001
**Occupation** (skilled)	3.03	1.05	5.02	8.942	0.003
**Marital Status** (unmarried)	4.46	2.20	6.72	14.967	<0.001
**Exposure to secondhand smoke** (yes)	4.26	2.56	5.95	24.198	<0.001
**Cigarette smoker** (yes)	1.85	0.48	3.21	7.046	0.008
**WTS status** (nonsmoker)	10.51	8.92	12.10	168.244	<0.001
**Survey round** (round 2)	6.96	5.73	8.19	122.637	<0.001
**WTP WL set** (novel)	6.68	6.19	7.17	717.051	<0.001

Multivariable linear regression models to study factors influencing the subscales of perceived efficacy of existing and novel WTP WLs showed that rural residence was significantly associated with all subscales, particularly with salience, affective reactions, and perceived harm **S3 and S4 Tables**. However, rural residence was inversely associated with credibility of the novel WTP WLs. Regarding the WTS status, being a nonsmoker was significantly associated with all subscales (except for relevance), specially with perceived harm and perceived behavioral control. Having a higher education was significantly associated with all subscales, specifically with perceived harm, depth of processing, and credibility of novel WTP WLs. The WTP WLs shown in survey round 2 were significantly associated with all subscales; compared with existing WTP WLs, the novel WTP WLs had higher associations with relevance, perceived harm, affective reactions, depth of processing, and perceived behavioral control **S3 and S4 Tables**.

## Discussion

This study presents one of the first attempts to measure the perceived efficacy of existing WTP WLs in comparison to novel enhanced generic and waterpipe-specific WLs. Furthermore, perceived efficacy of existing and novel WTP WLs was examined in waterpipe tobacco smokers and nonsmokers, as well as across various socio-demographic subgroups.

Participants rated novel WTP WLs with higher mean overall and subscale perceived efficacy scores than existing WLs, although both sets collectively scored modestly. The novel set was different from the existing set in three main aspects: the topical imagery content and its associated text, the WL size, and the pack design. These enhanced features were examined in this observational study collectively.

One of these enhanced features is the choice of imagery in the novel set, which featured proximal risks (e.g., dental and fetal effects) with elaborative waterpipe-specific text rather than information about long-term effects (e.g., cancer and ageing) and generic text displayed on the existing WTPs. This thematic choice was based on our previous qualitative study findings, where participants regarded WLs featuring proximal risks as most likely to be believable and acceptable [28]. Long-term events are usually viewed as more complex, unlikely, and unpredictable than proximal events [33]. In this study, we found that novel WTP WLs that featured proximal risks of WTS such as dental effects, fetal harm, and toxic contents of waterpipe tobacco had the highest participants’ perceived efficacy scores. This could be explained by the negative affective reactions and negative pack attitudes elicited by these topical contents [11]. WLs influence the perception of harm through two indirect pathways—affective reactions and cognitive processes; graphic images increase the concerns for health while text messages enhance the WL credibility [14]. In this study, waterpipe-specific novel WTP WLs were positively and independently associated with participants’ perceived efficacy scores. The specificity of novel WLs to WTS made the health education messages become clearer, and increased participants’ salience, relevance, affective reactions, depth of processing, and perceived harm; thus, improved the overall perceived efficacy of WTP WLs. Previous qualitative and quantitative studies that examined waterpipe-specific WLs (imagery and text) against text-only or generic WLs also confirmed the higher effectiveness of waterpipe-specific WLs and imagery content that depicted fetal harms due to WTS [24–26, 34].

The other two enhanced features are large pictorial WLs and plain packaging, which are recommended by the WHO FCTC [15, 16] because these enhancements maximize the salience and cognitive elaboration of health messages and WLs, render tobacco packaging and smoking less tempting, and reduce misperceptions about tobacco use harm [17–20, 35]. In line with this evidence, novel WLs in this study were particularly able to induce higher salience, affective reactions, and depth of processing, relative to the existing WTP WLs. Also, evidence from our previous qualitative study and from other developing countries supports this labelling policy [28, 36]. In addition, the different design features of WLs and WTP packaging in this study may explain the variant efficacy of both existing and novel WL sets. Observational [37] and experimental [38] research supports the favourable impact of plain packaging with larger WLs [39] on cognitive elaboration and behavioural changes [19]. The new WTPs in our study had a dark, uniform background; were not branded; and did not depict fruits or flavours. In addition, the novel WLs were larger so that the accompanying text was more readable and were placed at the top of the pack in the area that was previously occupied by colours and flavour information in the existing WL set. Plain packaging induces more visual attention towards WLs and away from branding and deceiving descriptors, especially among nonsmokers and light or non-established smokers [21]. Therefore, such packaging might reduce WTS uptake and increase the motivation to quit [28].

Both existing and novel WTP WL sets in our study presented modest perceived efficacy scores. Previous research found that smokers may reject fear-arousing graphic imagery and messages on WLs to lessen their feelings of threat [40, 41]. Reactance was considered the key to the perceived effectiveness by eliciting defensiveness and renunciation (cognitive processing) as well as annoyance and irritation (emotional reactance), even if WLs made individuals look away or avoid them [11]. Stronger reactions and aversiveness are linked to increased perception of harm, reading frequency of WLs, and subsequent quit attempts [13, 14, 41–43]. Differences in the participants’ perceived harm relative to waterpipe and cigarette smoking may have led to these modest scores as shown in previous studies [11]. Although the negative affective reactions were strong from both existing and novel WL sets, the modest effect of WLs in mediating health concerns, which were higher in nonsmokers than smokers, may be the cause of a lack in impact in perceived motivation of cessation behaviour. This finding is consistent with those of previous reports on the lower likelihood of behavioural changes among smokers in the short term after being exposed to WLs, although they rated warnings on plain packs or packs with pictorial WLs as more impactful [17, 38].

The nonsmokers in this study rated the WTP WLs with a higher perceived efficacy than the waterpipe tobacco smokers, specifically for perceived behavioral control. Being a nonsmoker also was independently and strongly associated with participants’ overall perceived efficacy scores. The effect of WLs on deterring smoking uptake remains scarcely studied. Nonsmokers are potential consumers of tobacco products and could benefit from customised warnings to reduce the intention to smoke [44]. Nonsmokers usually rated warnings as more effective than smokers [11], and seem to process warnings with increased perceived risk, which reinforces their continued nonsmoker status [14]. By contrast, tobacco smokers usually underestimate and misunderstand the smoking risks, and show unrealistic optimism regarding tobacco health hazards, especially lung cancer [45]. The WL ‘smoking causes lung cancer’ in this study received the lowest perceived efficacy ratings. This self-deception may be related to smokers’ false beliefs about safety from smoking health hazards if they just smoke for a few years, ability to quit smoking at any time, and attributing smoking-induced diseases to other factors [46]. Hence, waterpipe smokers have reported relatively lower ratings for perceived behavioral control than nonsmokers. Also, tobacco control efforts have long ignored WTS [5]. WTS is not emphasized as much as cigarettes in the media or by tobacco control efforts such as smoke-free policies, education interventions, or cessation services. This may have indirectly contributed to the misperceptions that WTS is less harmful than cigarette smoking. This unbalanced focus might explain why WLs that address WTS received relatively lower credibility ratings than other perceived efficacy subscales; participants might automatically associate smoking or the harms of smoking with cigarette smoking but not WTS. Also, we adjusted for the cigarette smoking status in our analyses because waterpipe smokers might be dual smokers and non-waterpipe smokers or “nonsmokers” in this study might be cigarette smokers; this variable was not accounted for in previous studies [24–26]. Hence, they may have been already exposed to the generic existing WLs, which are applied to cigarette and WTPs alike, during their usual smoking practices.

Rural residence and higher education were strongly associated with participants’ perceived efficacy scores. Participants who lived in rural areas in this study were mostly nonsmokers; this may partly explain why rural residence was highly associated particularly with salience, affective reactions, and perceived harm. On the other hand, individuals who received higher education may perceive the WLs as more convincing if accompanied by didactic text with facts [47], which may explain why the novel WLs were more believable among this subgroup. Participants who received more education reported higher ratings for perceived harm, depth of processing and credibility subscales, which is in line with previous findings, where communication of WTS risks and cognitive processing were several folds higher in this subgroup [23]. This might also explain why rural residence was inversely associated with credibility of the novel WTP WLs, as most rural residents had lower levels of education than their urban counterparts. Higher education was associated with higher WL effectiveness in a longitudinal study of 6011 adult smokers from six European countries [48].

### Strengths and limitations

In this study, we measured for the first time the perceived efficacy of existing WTP WLs in comparison to novel enhanced generic and waterpipe-specific WLs among waterpipe tobacco smokers and nonsmokers and across various socio-demographic groups. Our findings may provide basic evidence that future observational and experimental studies could use. However, the study presented certain limitations. First, the cross-sectional study design limits the ability to test whether these findings will translate into extended behavioural outcomes past the study period. In this study, we intended to measure only instant efficacy; therefore, we did not resort to a longitudinal design. The purposive sampling does not support external validity of study results. Nonetheless, the large sample size allowed for sufficient observations among the compared subgroups and may compensate for potential sources of bias in the results.

Second, the topical content of the imagery that was used in this study was different in both sets and in both study rounds, which would be expected because our research spanned over 2 years (equivalent to the WL set rotation period). We used suitable statistical analysis methods to account for within subject correlation and repeated measures data and to identify factors and individual WLs that were independently associated with participants’ perceived efficacy of WTP WLs. Also, novel WLs were designed based on the WHO FCTC guidelines, the best available evidence on WTS hazards, and our qualitative research conducted in parallel guided the design of the graphic warnings of the novel WL set of the second study round.

Third, in both survey rounds, all participants rated the perceived efficacy of an existing WL first before exposure to a novel WL. This order of viewing WTP WLs was chosen to ensure that the data interviewers carried out this task consistently; additional instructions for randomizing participants’ exposure to different WTP WL sets in the field may have added another layer of complexity for the data interviewers during their field work. This is because the study was not carried out in a controlled environment (participants were met in different locations such as workplaces, cafes, homes, university) and the face-interview took approximately 25 minutes to complete. This order of participants’ exposure to WTP WLs has some limitations. Although novel WTP WLs had higher perceived efficacy scores than existing WLs, the difference between the two sets was modest. This might be attributed to always viewing the existing WL first, thus diminishing the effect of viewing the novel WLs afterwards. However, this order effect may not have necessarily biased perceived efficacy ratings of the novel set towards lower scores. Previous research showed that the order in which individuals are influenced by messages is mediated through two main effects, either primacy or recency [49]. The former induces higher message influence after the initial communication and the latter assumes that the final effects have higher message influence. Both order effects could occur; it depends on individual factors such as interest and motivation [49].

Fourth, almost all published theories are from cigarette pack research, which may not be applicable to WTPs because of different exposure rates in real life. Compared with the experience of the smokers viewing cigarette packs several times a day, the experience tested here simulated realistic conditions in which waterpipe smokers are exposed to the WTP fewer times or not at all if they did not prepare the hagar themselves. Compared with experimental cigarette pack studies and previous waterpipe tobacco studies, which presented participants with a brief exposure to WLs on computer screens, the participants in our study handled a real WTP with a novel WL so that the stimuli were comparable to existing WLs they may encounter in real life.

Lastly, this study included an experimental element in which new pack designs and new WLs were tested according to an observational study design. This design was exploratory; it was not intended as an experimental study; hence, participants were not randomized and there were no control groups. Participants viewed only one WL from each set to reduce the introduction of disorienting stimuli that occurs when viewing many warnings in the same setting [50]. Moreover, the participants were presented with warnings in a random manner so that each imagery was equally tested in the study sample. The enhanced features of novel WLs were examined in this observational study collectively. We tested several variables to measure perceived efficacy. If we manipulated only one variable between the existing and novel WL sets, we could have been able to distinguish single effects of each of the pack designs, waterpipe-specific imagery contents and texts. However, the approach we adopted helped in testing the combined effect of the three proposed changes in close to real-life conditions, which could inform WTP labelling policy more practically than an experimental design that is intended to single-out the individual effects of each change in controlled conditions.

Whether experimental or observational designs are the best method of gathering evidence on the actual impact of WLs remains controversial. Observational studies typically have more external validity, especially if they employ follow-up investigations of WTS initiation or cessation behaviours post-WL introduction and the effects on a population-level. Researchers in favour of experimental designs argue that it is difficult to isolate the effects of WLs on smoking behaviour when other tobacco control policies are implemented concurrently; thus, evidence of the contributory impact of WLs obtained by experiments may be stronger by isolating the effects on single outcomes [11]. However, the currently implemented tobacco control policies in Egypt seem to be ineffective in reducing WTS; therefore, we can presume that the effects from this study are isolated. Further observational and experimental designs can build on these points for more concrete WTP WL policy configurations.

### Conclusions

To inform the WTP labelling policy, we measured for the first time the efficacy of the existing WTP WL set in Egypt, which depicts fruits and flavour information, on WTPs compared with that of a novel set with larger WLs, plain packaging, and more waterpipe-specific graphic imagery and text. We examined the possible effects of these sets among waterpipe smokers and nonsmokers and across socio-demographic groups.

The novel WL set ratings were significantly higher than those for the existing set for all efficacy measures, although both sets collectively scored modestly. Relative to the existing WTP WLs, novel WLs were particularly able to induce higher salience, affective reactions, and depth of processing. Relative to the generic novel WTP WLs, waterpipe-specific WLs induced higher relevance, perceived harm, and affective reactions. Nonsmokers scored higher than waterpipe tobacco smokers, specifically for perceived behavioral control. WTP WLs featuring proximal risks, toxic contents, or fetal harm were strongly associated with participants’ perceived efficacy scores. Among other independent factors, rural residence, higher education, being a nonsmoker, and waterpipe-specific novel WTP WLs were highly associated with participants’ perceived efficacy scores.

Waterpipe-specific WLs on plain WTPs that feature proximal risks need to be developed in conjunction with awareness raising campaigns on WTS harms to reinforce the credibility of WTP WLs. Evidence-based WL content and designs that address different population subgroups must be adopted. Our findings suggest the proposed WTP WL enhancements by the Tobacco Control Unit may support a more effective WTP labelling policy within a comprehensive waterpipe-specific tobacco control framework.

## Supporting information

S1 Table Usage rates of existing and novel WTP WLs at each survey round, Egypt, 2015–2017 (N = 2014). (DOCX)

S2 Table Total perceived efficacy scores of existing and novel WTP WLs by participants’ background characteristics, Egypt, 2015–2017 (n = 2014). (DOCX)

S3 Table Multivariable linear regression models for factors associated with perceived efficacy subscales of existing WTP WLs, Egypt, 2015–2017 (n = 2014). (DOCX)

S4 Table Multivariable linear regression models for factors associated with perceived efficacy subscales of novel WTP WLs, Egypt, 2015–2017 (n = 2014). (DOCX)

S1 Appendix Contains the questions used in the current study and S1–S4 Tables. (DOCX)
