# Supplementary material for: Perceived efficacy of existing waterpipe tobacco warning labels versus novel enhanced generic and waterpipe-specific sets
Source: PLoS One. 2021 Jul 27;16(7):e0255244. doi: 10.1371/journal.pone.0255244 (PMC8315518; doi:10.1371/journal.pone.0255244)
Supplement: S1 Table — (DOCX) [file pone.0255244.s001.docx]

**S1 Table. Usage rates of existing and novel WTP WLs at each survey round, Egypt, 2015-2017 (N=2014)**

| **Existing WTP WLs** | | | **Novel WTP WLs** | | |
| --- | --- | --- | --- | --- | --- |
| **Survey round 1** | **N=1015** | | **Survey round 1** | **N=1015** | |
|  | **n** | **%** |  | **n** | **%** |
| Smoking causes lung cancer | 222 | 21.9 | Smoking causes blindness | 214 | 21.1 |
| Smoking causes mouth cancer | 275 | 27.1 | Smoking destroys teeth and gums | 219 | 21.6 |
| Smoking causes throat cancer | 292 | 28.8 | Smoking in pregnancy harms the fetus | 306 | 30.1 |
| Smoking causes face cancer | 226 | 22.2 | Smoking causes blood vessel clotting | 276 | 27.2 |
| **Survey round 2** | **N=999** | | **Survey round 2** | **N=999** | |
|  | **n** | **%** |  | **n** | **%** |
| Smoking burns body organs and causes cancers and cardiac diseases | 248 | 24.8 | Waterpipe smoking causes teeth and gum decay | 240 | 24.0 |
| Cigarettes contain ‘DDT’ that is used in killing insects | 265 | 26.6 | Waterpipe smoking exposes you to toxins like that used to kill insects | 310 | 31.0 |
| Smoking leads to early aging and disability | 248 | 24.8 | Waterpipe smoking during pregnancy harms the fetus | 251 | 25.1 |
| Passive smoking harms the fetus and may lead to reduced weight at birth | 238 | 23.8 | Waterpipe smoking causes blood vessel clotting | 198 | 19.9 |
