# Supplementary material for: Perceived efficacy of existing waterpipe tobacco warning labels versus novel enhanced generic and waterpipe-specific sets
Source: PLoS One. 2021 Jul 27;16(7):e0255244. doi: 10.1371/journal.pone.0255244 (PMC8315518; doi:10.1371/journal.pone.0255244)
Supplement: S2 Table — (DOCX) [file pone.0255244.s002.docx]

**S2 Table. Total perceived efficacy scores of existing and novel WTP WLs by participants' background characteristics, Egypt, 2015-2017 (n=2014)**

|  | **Total perceived efficacy score** | | | | | |
| --- | --- | --- | --- | --- | --- | --- |
|  | **Existing WTP WLs** | **F** | **p-value^a^** | **Novel WTP WLs** | **F** | **p-value^a^** |
|  | **Mean (SE)** |  |  | **Mean (SE)** |  |  |
| **Age group** | | | | | | |
| 18-24 | 55.4 (0.8) | 13.663 | <0.001 | 61.8 (0.6) | 6.339 | <0.001 |
| ≥25 | 51.7 (0.6) |  |  | 58.5 (0.5) |  |  |
| **Gender** | | | | | | |
| Female | 40.5 (1.6) | 71.039 | <0.001 | 51.7 (1.6) | 45.486 | <0.001 |
| Male | 54.4 (0.5) |  |  | 60.6 (0.4) |  |  |
| **Residence** | | | | | | |
| Urban | 34.9 (0.7) | 1723.268 | <0.001 | 49.8 (0.7) | 178.762 | <0.001 |
| Rural | 65.5 (0.4) |  |  | 66.6 (0.4) |  |  |
| **Education** | | | | | | |
| University/vocational | 48.2 (0.8) | 87.697 | <0.001 | 58.8 (0.6) | 38.613 | 0.027 |
| Less than university/vocational | 57.3 (0.6) |  |  | 60.6 (0.5) |  |  |
| **Occupation** | | | | | | |
| Unskilled | 48.1 (0.8) | 102.032 | <0.001 | 57.6 (0.6) | 24.632 | <0.001 |
| Skilled | 57.8 (0.6) |  |  | 61.8 (0.5) |  |  |
| **Marital Status** | | | | | | |
| Unmarried | 54.4 (0.8) | 4.661 | 0.031 | 61.7 (0.7) | 0.003 | <0.001 |
| Married | 52.2 (0.6) |  |  | 58.6 (0.5) |  |  |
| **Exposure to secondhand smoke** | | | | | | |
| No | 43.5 (0.8) | 245.922 | <0.001 | 54.9 (0.7) | 41.283 | <0.001 |
| Yes | 58.6 (0.5) |  |  | 62.6 (0.5) |  |  |
| **WTS status** | | | | | | |
| Current waterpipe smoker | 50.4 (0.6) | 86.858 | <0.001 | 57.5 (0.5) | 15.719 | <0.001 |
| Nonsmoker | 60.6 (0.9) |  |  | 66.1 (0.7) |  |  |
| **Cigarette smoker** | | | | | | |
| No | 51.4 (0.6) | 7.653 | <0.001 | 59.1 (0.5) | 0.062 | 0.043 |
| Yes | 55.9 (0.8) |  |  | 60.8 (0.7) |  |  |
| **Survey round** | | | | | | |
| Round 1 | 49.9 (0.7) | 42.417 | <0.001 | 56.0 (0.5) | 0.832 | <0.001 |
| Round 2 | 56.2 (0.7) |  |  | 63.5 (0.6) |  |  |

SE: Standard error

**^a^** Independent Samples t-test
