# Supplementary material for: Perceived efficacy of existing waterpipe tobacco warning labels versus novel enhanced generic and waterpipe-specific sets
Source: PLoS One. 2021 Jul 27;16(7):e0255244. doi: 10.1371/journal.pone.0255244 (PMC8315518; doi:10.1371/journal.pone.0255244)
Supplement: S3 Table — (DOCX) [file pone.0255244.s003.docx]

**S3 Table. Multivariable linear regression models for factors associated with perceived efficacy subscales of existing WTP WLs, Egypt, 2015-2017 (n=2014)**

|  | **Salience** | **p- value** | **Credibility** | **p- value** | **Relevance** | **p- value** | **Perceived harm** | **p- value** | **Affective reactions** | **p- value** | **Depth of processing** | **p- value** | **Perceived behavioural control** | **p- value** |
| --- | --- | --- | --- | --- | --- | --- | --- | --- | --- | --- | --- | --- | --- | --- |
|  | **β (95% CI)** |  | **β (95% CI)** |  | **β (95% CI)** |  | **β (95% CI)** |  | **β (95% CI)** |  | **β (95% CI)** |  | **β (95% CI)** |  |
| **Adjusted R2** | 0.605 |  | 0.138 |  | 0.318 |  | 0.36 |  | 0.643 |  | 0.427 |  | 0.286 |  |
| **Age** (≥25) | 2.33  (-0.54, 5.21) | 0.111 | 3.57  (1.25, 5.88) | 0.003 | 0.47  (-2.12, 3.07) | 0.720 | 3.86  (1.12, 6.61) | 0.006 | 2.70  (0.19, 5.22) | 0.035 | 2.65  (0.68, 4.63) | 0.008 | 3.29  (0.26, 6.3) | 0.033 |
| **Gender** (male) | -0.82  (-4.31, 2.66) | 0.643 | -3.43  (-6.23,-0.62 ) | 0.017 | 0.78  (-2.36, 3.93) | 0.626 | -1.07  (-4.40, 2.26) | 0.528 | -2.58  (-5.63 ,0.47) | 0.098 | -1.93  (-4.32, 0.47) | 0.114 | -0.45  (-4.12, 3.22) | 0.808 |
| **Residence** (rural) | 50.48  (48.01, 52.95) | <0.001 | 11.49  (9.50, 13.48 ) | <0.001 | 20.90  (18.67, 23.14) | <0.001 | 26.42  (24.06, 28.79) | <0.001 | 48.01  (45.84, 50.17) | <0.001 | 23.10  (21.40, 24.7) | <0.001 | 17.65  (15.0520.26 | <0.001 |
| **Education** (university/vocational) | 3.91  (1.14, 6.69) | 0.006 | 5.89  (3.66, 8.12) | <0.001 | 5.23  (2.72, 7.73) | <0.001 | 6.17  (3.5, 8.82) | <0.001 | 5.54  (3.11 ,7.97) | <0.001 | 5.50  (3.62, 7.40) | <0.001 | 6.18  (3.26, 9.09 | <0.001 |
| **Occupation** (skilled) | 1.16  (-1.45, 3.77) | 0.384 | 2.91  (0.81, 5.01) | 0.007 | 5.01  (2.66, 7.37) | <0.001 | 2.53  (0.04, 5.03) | 0.047 | 3.83  (1.51, 6.12) | 0.001 | 2.98  (1.13, 4.77) | 0.001 | 3.47  (0.72, 6.22) | 0.013 |
| **Marital Status** (unmarried) | 5.39  (2.46, 8.32) | <0.001 | 3.12  (0.77, 5.48) | 0.009 | 1.69  (-0.95, 4.33) | 0.209 | 6.45  (3.65, 9.24) | <0.001 | 5.36  (2.80, 7.92) | <0.001 | 3.25  (1.24, 5.26) | 0.002 | 7.63  (4.54,10.7) | <0.001 |
| **Exposure to secondhand smoke** (yes) | 7.35  (5.07, 9.63) | <0.001 | 3.16  (1.33, 5.00) | 0.001 | 6.94  (4.89, 9.00) | <0.001 | 5.75  (3.58, 7.93) | <0.001 | 7.03  (5.04, 9.02) | <0.001 | 5.04  (3.47, 6.60) | <0.001 | 2.49  (0.09, 4.88) | 0.042 |
| **WTS status** (nonsmoker) | 7.65  (5.36, 9.94) | <0.001 | 8.28  (6.43, 10.12) | <0.001 | -1.48  (-3.54, 0.59) | 0.162 | 15.03  (12.85,17.22) | <0.001 | 9.22  (7.21, 11.22) | <0.001 | 8.15  (6.58, 9.73) | <0.001 | 24.18  (21.77, 26.59) | <0.001 |
| **Cigarette smoker** (yes) | 2.78  (0.78, 4.78) | 0.006 | 0.47  (-1.14, 2.08) | 0.570 | 1.80  (-0.01, 3.60) | 0.051 | 2.15  (0.24, 4.06) | 0.028 | 2.82  (1.07, 4.5) | 0.002 | 1.41  (0.03, 2.78) | 0.045 | 4.43  (2.33, 6.54) | <0.001 |
| **Survey round** (round 2) | 8.01  (6.10, 9.91) | <0.001 | 2.27  (0.74, 3.80) | 0.004 | 8.99  (7.27, 10.71) | <0.001 | 6.43  (4.61, 8.25) | <0.001 | 6.21  (4.54, 7.88) | <0.001 | 5.97  (4.66 ,7.28) | <0.001 | 6.55  (4.54, 8.55) | <0.001 |
| **R2** | 0.779 |  | 0.377 |  | 0.567 |  | 0.603 |  | 0.803 |  | 0.655 |  | 0.538 |  |
