# Supplementary material for: Perceived efficacy of existing waterpipe tobacco warning labels versus novel enhanced generic and waterpipe-specific sets
Source: PLoS One. 2021 Jul 27;16(7):e0255244. doi: 10.1371/journal.pone.0255244 (PMC8315518; doi:10.1371/journal.pone.0255244)
Supplement: S4 Table — (DOCX) [file pone.0255244.s004.docx]

**S4 Table. Multivariable linear regression models for factors associated with perceived efficacy subscales of novel WTP WLs, Egypt, 2015-2017 (n=2014)**

|  | **Salience** | **p- value** | **Credibility** | **p- value** | **Relevance** | **p- value** | **Perceived harm** | **p- value** | **Affective reactions** | **p- value** | **Depth of processing** | **p- value** | **Perceived behavioural control** | **p- value** |
| --- | --- | --- | --- | --- | --- | --- | --- | --- | --- | --- | --- | --- | --- | --- |
|  | **β**  **(95% CI)** |  | **β**  **(95% CI)** |  | **β**  **(95% CI)** |  | **β**  **(95% CI)** |  | **β**  **(95% CI)** |  | **β (95% CI)** |  | **β (95% CI)** |  |
| **Adjusted R2** | 0.455 |  | 0.098 |  | 0.0143 |  | 0.232 |  | 0.511 |  | 0.157 |  | 0.192 |  |
| **Age** (≥25 years) | 1.14  (-1.69, 3.90) | 0.430 | 2.96  (0.49, 5.42) | 0.019 | -0.01  (-2.83, 2.81) | 0.995 | 2.14  (-0.63, 4.91) | 0.129 | 1.86  (-0.57, 4.30) | 0.134 | 1.13  (-0.90, 3.15) | 0.276 | 1.89  (-1.22, 5.01) | 0.234 |
| **Gender** (male) | 5.30  (1.86, 8.73) | 0.003 | 2.07  (-0.92, 5.06) | 0.175 | 2.68  (-0.7 ,6.10) | 0.124 | -0.48  (-3.84, 2.88) | 0.779 | -0.05  (-3.01, 2.9) | 0.972 | 2.37  (-0.09, 4.83) | 0.059 | 0.04  (-3.75, 3.82) | 0.984 |
| **Residence** (rural) | 36.32  (33.88, 38.76) | 0.000 | -7.41  (-9.53, -5.29) | 0.000 | 9.46  (7.04, 11.89) | <0.001 | 17.63  (15.24, 20.01) | <0.001 | 35.08  (32.99, 37.18) | <0.001 | 8.90  (7.15, 10.64) | <0.001 | 6.10  (3.42, 8.78) | <0.001 |
| **Education** (university/vocational) | 3.96  (1.23, 6.69) | 0.005 | 6.48  (4.10, 8.86) | 0.000 | 5.20  (2.4, 7.92) | <0.001 | 8.15  (5.48, 10.83) | <0.001 | 6.73  (4.37, 9.08) | <0.001 | 7.48  (5.52, 9.43) | <0.001 | 6.34  (3.33, 9.35) | <0.001 |
| **Occupation** (skilled) | 1.07  (-1.51, 3.64) | 0.416 | 2.63  (0.39, 4.87) | 0.022 | 4.68  (2.12, 7.24) | <0.001 | 3.05  (0.53, 5.56) | 0.018 | 3.01  (0.79, 5.22) | 0.008 | 3.09  (1.25, 4.94) | <0.001 | 2.57  (-0.27, 5.40) | 0.076 |
| **Marital status**  (unmarried) | 4.95  (2.07, 7.84) | 0.001 | 2.91  (0.40, 5.4) | 0.023 | 1.45  (-1.42, 4.32) | 0.322 | 4.42  (1.6, 7.24) | 0.002 | 5.06  (2.57, 7.54) | <0.001 | 2.31  (0.2, 4.37) | 0.028 | 6.33  (3.16, 9.52) | <0.001 |
| **Exposure to secondhand smoke** (yes) | 4.88  (2.64, 7.12) | 0.000 | 1.26  (-0.70, 3.21) | 0.207 | 3.68  (1.44, 5.91) | <0.001 | 3.52  (1.33, 5.72) | 0.002 | 5.56  (3.63, 7.49) | <0.001 | 2.76  (1.16, 4.36) | 0.001 | 2.02  (-0.46, 4.49) | 0.110 |
| **WTS status** (nonsmoker) | 3.94  (1.6, 6.19) | 0.001 | 5.50  (3.54, 7.40) | 0.000 | -3.62  (-5.87, -1.38) | <0.001 | 13.45  (11.24, 15.66) | <0.001 | 6.91  (4.97, 8.85) | <0.001 | 5.22  (3.60, 6.84) | <0.001 | 22.35  (19.87, 24.84) | <0.001 |
| **Cigarette smoker** (yes) | 0.44  (-1.53, 2.41) | 0.660 | -1.49  (-3.20, 0.23) | 0.089 | 0.96  (-1.00 ,2.93) | 0.335 | 1.03  (-0.90, 2.91) | 0.297 | 2.34  (0.64, 4.04) | 0.007 | -0.73  (-2.14, 0.68) | 0.312 | 3.18  (1.01, 5.35) | 0.004 |
| **Survey round** (round 2) | 6.02  (4.14, 7.90) | 0.000 | 2.32 (0.69, 3.96) | 0.005 | 10.74  (8.87, 12.61) | <0.001 | 8.13  (6.29, 9.97) | <0.001 | 7.74  (6.12, 9.36) | <0.001 | 6.69  (5.34, 8.03) | <0.001 | 7.89  (5.82, 9.96) | <0.001 |
| **R2** | 0.676 |  | 0.320 |  | 0.384 |  | 0.486 |  | 0.716 |  | 0.402 |  | 0.443 |  |
