# Supplementary material for: Perceived efficacy of existing waterpipe tobacco warning labels versus novel enhanced generic and waterpipe-specific sets
Source: PLoS One. 2021 Jul 27;16(7):e0255244. doi: 10.1371/journal.pone.0255244 (PMC8315518; doi:10.1371/journal.pone.0255244)
Supplement: S1 Appendix — (DOCX) [file pone.0255244.s005.docx]

**S1 Appendix**

**Questions on perceived efficacy of existing and novel WTP WLs and corresponding subscales**

| **Perceived efficacy**  **subscale** | **Existing WTP WL code _____**(data interviewer records code for one of the four existing WTP WLs)  Instructions to participant: Please look closely at the pictorial health warning on this waterpipe tobacco pack before answering the following questions:  On a scale of 1 to 10, please describe whether this pictorial health warning:  1= not at all and 10= very likely | |
| --- | --- | --- |
| **Salience** | grabs your attention | 1 2 3 4 5 6 7 8 9 10 |
| **Credibility** | is believable | 1 2 3 4 5 6 7 8 9 10 |
| **Relevance** | is relevant to you | 1 2 3 4 5 6 7 8 9 10 |
| **Perceived harm** | would make you more concerned about the health risks of waterpipe tobacco smoking | 1 2 3 4 5 6 7 8 9 10 |
| **Affective reactions** | is surprising | 1 2 3 4 5 6 7 8 9 10 |
|  | is frightening | 1 2 3 4 5 6 7 8 9 10 |
|  | is unpleasant | 1 2 3 4 5 6 7 8 9 10 |
|  | would make you want to avoid looking at it by covering it up for example | 1 2 3 4 5 6 7 8 9 10 |
| **Depth of processing** | makes you want to look at it or read it closely | 1 2 3 4 5 6 7 8 9 10 |
|  | is understandable | 1 2 3 4 5 6 7 8 9 10 |
|  | accurately depicts risks of waterpipe tobacco smoking to your health and the health of those surrounding you | 1 2 3 4 5 6 7 8 9 10 |
| **Perceived behavioural control** | | |
| For waterpipe tobacco smokers only | would stop you from smoking waterpipe tobacco if you were about to smoke one | 1 2 3 4 5 6 7 8 9 10 |
|  | would make you reduce the number of waterpipe tobacco hagar (portion) that you smoke | 1 2 3 4 5 6 7 8 9 10 |
|  | would make you want to quit waterpipe tobacco smoking | 1 2 3 4 5 6 7 8 9 10 |
| For nonsmokers only | would help prevent you from starting to smoke waterpipe tobacco | 1 2 3 4 5 6 7 8 9 10 |
| **Perceived efficacy**  **subscale** | **Novel WTP WL code ______**(data interviewer records code for one of the four existing WTP WLs)  Instructions to the same participant: Please look closely at the pictorial health warning on this waterpipe tobacco pack before answering the following questions:  On a scale of 1 to 10, please describe whether this pictorial health warning:  1= not at all and 10= very likely | |
| **Salience** | grabs your attention | 1 2 3 4 5 6 7 8 9 10 |
| **Credibility** | is believable | 1 2 3 4 5 6 7 8 9 10 |
| **Relevance** | is relevant to you | 1 2 3 4 5 6 7 8 9 10 |
| **Perceived harm** | would make you more concerned about the health risks of waterpipe tobacco smoking | 1 2 3 4 5 6 7 8 9 10 |
| **Affective reactions** | is surprising | 1 2 3 4 5 6 7 8 9 10 |
|  | is frightening | 1 2 3 4 5 6 7 8 9 10 |
|  | is unpleasant | 1 2 3 4 5 6 7 8 9 10 |
|  | would make you want to avoid looking at it by covering it up for example | 1 2 3 4 5 6 7 8 9 10 |
| **Depth of processing** | makes you want to look at it or read it closely | 1 2 3 4 5 6 7 8 9 10 |
|  | is understandable | 1 2 3 4 5 6 7 8 9 10 |
|  | accurately depicts risks of waterpipe tobacco smoking to your health and the health of those surrounding you | 1 2 3 4 5 6 7 8 9 10 |
| **Perceived behavioural control** | | |
| For waterpipe tobacco smokers only | would stop you from smoking waterpipe tobacco if you were about to smoke one | 1 2 3 4 5 6 7 8 9 10 |
|  | would make you reduce the number of waterpipe tobacco hagar (portion) that you smoke | 1 2 3 4 5 6 7 8 9 10 |
|  | would make you want to quit waterpipe tobacco smoking | 1 2 3 4 5 6 7 8 9 10 |
| For nonsmokers only | would help prevent you from starting to smoke waterpipe tobacco | 1 2 3 4 5 6 7 8 9 10 |
